# Neutrophil‐to‐lymphocyte ratio can specifically predict the severity of hypertriglyceridemia‐induced acute pancreatitis compared with white blood cell

**DOI:** 10.1002/jcla.22839

**Published:** 2019-02-08

**Authors:** Li Huang, Congying Chen, Lijuan Yang, Rong Wan, Guoyong Hu

**Affiliations:** ^1^ Department of Gastroenterology, Shanghai General Hospital Shanghai Jiao Tong University School of Medicine Shanghai China

**Keywords:** acute pancreatitis, alcoholic, gallstones, hypertriglyceridemia, neutrophil‐to‐lymphocyte ratio

## Abstract

**Objectives:**

We aimed to evaluate the values of neutrophil‐to‐lymphocyte ratio (NLR) and white blood cell (WBC) in predicting severity of acute pancreatitis (AP) with different etiologies.

**Methods:**

We compared NLR and WBC levels in patients with different etiologies and AP severity. The optimal cutoff value for them to predict severe acute pancreatitis (SAP) was determined by receiver operating characteristic (ROC) curve analysis.

**Results:**

Both NLR and WBC were elevated in patients with SAP. After subgrouping AP by etiology, NLR was predictive of SAP only in hypertriglyceridemia‐induced AP (HTG‐AP), while WBC could effectively predict severity in both gallstone and HTG‐AP. The best cutoff value of WBC for predicting SAP in gallstone AP patients was 12.81 × 10^9^/L, with sensitivity and specificity of 78.9% and 70.2%. The best cutoff value for NLR and WBC to differentiate HTG‐SAP was more than 5.88 and 15.89 × 10^9^/L, respectively, with sensitivity and specificity of 87% and 50% for NLR and 56.5% and 75.76% for WBC.

**Conclusions:**

Our study firstly demonstrated that NLR selectively played a role in HTG‐AP, while WBC could predict the severity of both gallstone and HTG‐AP. Furthermore, we firstly elucidated that NLR was more sensitive and accurate in judging the severity of HTG‐AP compared with WBC.

## 1 INTRODUCTION

Acute pancreatitis (AP) is a common digestive system emergency, of which severe acute pancreatitis (SAP) has a rapid onset and high mortality due to systemic inflammatory response syndrome.^1^ Thus, early identification of patients with severe inclination and early effective intervention may improve prognosis of the patients. This indicates the importance of prediction of SAP.^2^

A number of severity scoring systems have been constructed for early prediction of SAP, for example, the Acute Physiology and Chronic Health Evaluation (APACHEII) system, the Ranson score, and the Bedside Index for Severity in Acute Pancreatitis (BISAP) score.^3^ However, due to their complex variables and long elapsed time, simplified serum markers are needed.

During recent years, neutrophil‐to‐lymphocyte ratio (NLR) has been proved of great value with a significant correlation with systemic inflammation reaction in several autoimmune diseases.^4^, ^5^ Because NLR can be easily obtained at low cost through an automatic hematology analyzer, many research groups have further confirmed the value of NLR in predicting disease severity and worse clinical outcomes in a variety of diseases, including cerebrovascular diseases,^6^, ^7^, ^8^ cardiovascular diseases,^9^ and neoplasm.^10^ Besides, cumulative evidence has suggested that NLR was associated with AP and better than other serum markers in predicting severity and prognosis of AP. However, there was a lack of data regarding their utilities and comparison in the development of AP with different etiologies. The aim of this study was to explore the value of NLR in determining the severity of AP with different causes compared with white blood cell (WBC).

## 2 MATERIALS AND METHODS

### 2.1 Patients

We recruited 268 AP patients admitted to digestive department of our hospital from January 2012 to January 2017. AP was diagnosed based on patients’ clinical symptoms, radiographic examination, and laboratory results. Disease severity was assessed using Atlanta typing 2012.^11^ The most three common causes of AP are gallstone, hypertriglyceridemia, and alcohol.^1^, ^12^ There were 223 cases with mild acute pancreatitis (MAP) and 45 cases with moderately severe acute pancreatitis (MSAP) or SAP, of which 123 were gallstone AP, 56 were alcoholic AP, and 89 were hypertriglyceridemia‐induced AP (HTG‐AP). Because the sample size was not big enough, MSAP was also classified as SAP in our study. Exclusion criteria are as follows: (a) other causes induced AP; (b) acute exacerbation of chronic pancreatitis; (c) tumor induced AP; (d) AP onset was more than 24 hours when at hospital; and (e) missing data were available.

### 2.2 Data collection

Peripheral blood samples were obtained within 24 hours after AP onset and detected by a full‐automatic hemolytic analyzer. WBC, neutrophil, and lymphocyte counts were obtained, and NLR was calculated. Demographic variables (including age and gender) were also included.

### 2.3 Statistical analysis

Continuous variables were presented as mean ± standard deviation. Frequency and percentage (%) were used to describe categorical data. The analysis of variance and *t* test were applied in comparing the intergroup difference of measurement data. The optimal cutoff values of NLR and WBC were determined by ROC curve analysis. Test which has the larger AUC has the better diagnostic value. A *P*‐value <0.05 was considered statistically significant. All analyses were performed using SPSS 17.0 Software (SPSS, Inc., Chicago, IL, USA).

## 3 RESULTS

### 3.1 Clinical characteristics

A total of 268 patients with AP were enrolled, consisted of 140 males and 128 females with an average age of 55 years old (range: 28‐75). We next classified patients according to the etiology, of which gallstone accounted for 45.90% (n = 123), alcohol 20.90% (n = 56), and hyperlipidemia 33.21% (n = 89). According to the Atlanta typing 2012, SAP had a proportion of 16.8% (n = 45). There were no statistical differences in age and sex between SAP group and MAP group. Thus, we excluded age and sex as latent impurity interferon of SAP. Patients with SAP mainly came from HTG‐AP and gallstone AP groups (Table 1). In our study, SAP accounted for 15.45%, 5.36%, and 25.84% in gallstone AP, alcoholic AP, and HTG‐AP, respectively (Table 2).

**Table 1:** Demographic and etiologic characteristics by severity

Severity	Male (no.)	Female (no.)	Age (y)	Gallstone (%)	Alcohol (%)	Hyperlipidemia (%)
MAP	116	107	54.71 ± 21.03	46.64	23.77	29.60
SAP	24	21	56.44 ± 28.12	42.22	6.67	51.11
*P*	0.291		0.733			

MAP, mild acute pancreatitis; no., number; SAP, severe acute pancreatitis; y, years.

**Table 2:** Proportion of severity of pancreatitis by etiology

Etiology	Severity	Number	Percentage
Gallstone	MAP	104	84.55
	SAP	19	15.45
Alcohol	MAP	53	94.64
	SAP	3	5.36
Hyperlipidemia	MAP	66	74.16
	SAP	23	25.84

MAP, mild acute pancreatitis; SAP, severe acute pancreatitis.

### 3.2 NLR and WBC Levels within 24 hours after AP Onset

White blood cell counts (14.53 ± 4.76 vs 12.44 ± 4.52, 10.90 ± 4.13 × 10^9^/L) were significantly higher in the HTG‐AP group than in the gallstone (*P* = 0.001) and alcoholic group (*P* = 0.000). The gallstone AP group showed significantly higher NLR levels (10.50 ± 8.28 vs 6.06 ± 5.41, *P* = 0.000) than the alcoholic group. NLR did not differ significantly between the gallstone AP group and HTG‐AP group (10.50 ± 8.28 vs 8.70 ± 7.12, *P* = 0.082; Table 3).

**Table 3:** NLR and WBC levels within 24 h after AP onset by etiology

Etiology	Number	NLR	WBC (10^9^/L)
Gallstone	123	10.50 ± 8.28	12.44 ± 4.52
Alcohol	56	6.06 ± 5.41	10.90 ± 4.13
Hyperlipidemia	89	8.70 ± 7.12	14.53 ± 4.76

AP, acute pancreatitis; NLR, neutrophil‐to‐lymphocyte ratio; WBC, white blood cell.

The mean levels of NLR and WBC in patients with SAP were significantly higher than those in the MAP group (12.24 ± 8.37 vs 8.32 ± 7.24, *P* = 0.001; 16.17 ± 5.00 vs 12.14 ± 4.35 × 10^9^/L, *P* = 0.000, respectively; Table 4). We next performed subgroup analysis according to etiology of AP. For gallstone AP, there was no significant difference in NLR between MAP and SAP (10.08 ± 8.52 vs 12.80 ± 6.56, *P* = 0.189). On the contrary, the difference in WBC was obvious (11.8 ± 4.39 vs 15.92 ± 3.59, *P* = 0.000). In the HTG‐AP group, NLR and WBC levels were both significantly higher in SAP (12.41 ± 10.01 vs 7.41 ± 5.44, *P* = 0.004; 16.5 ± 5.93 vs 13.85 ± 4.11 × 10^9^/L, *P* = 0.020, respectively) than in MAP. In particular, the difference in NLR was more obvious than in WBC. In the alcoholic AP group, our study showed neither NLR nor WBC could be regarded as indicators of the disease severity (Table 5).

**Table 4:** Comparison of NLR and WBC Levels within 24 h after AP onset by severity

Severity	Number	NLR	WBC(10^9^/L)
MAP	223	8.32 ± 7.24	12.14 ± 4.35
SAP	45	12.24 ± 8.37	16.17 ± 5.00
*P*		0.001	0.000

AP, acute pancreatitis; MAP, mild acute pancreatitis; NLR, neutrophil‐to‐lymphocyte ratio; SAP, severe acute pancreatitis; WBC, white blood cell.

**Table 5:** Comparison of NLR and WBC levels between MAP and SAP by etiology

Etiology	Severity	NLR	WBC (10^9^/L)
Gallstone	MAP	10.08 ± 8.52	11.8 ± 4.39
	SAP	12.80 ± 6.56	15.92 ± 3.59
	*P*	0.189	0
Alcohol	MAP	5.98 ± 5.52	10.66 ± 3.91
	SAP	7.41 ± 3.50	15.21 ± 6.56
	*P*	0.661	0.063
Hyperlipidemia	MAP	7.41 ± 5.44	13.85 ± 4.11
	SAP	12.41 ± 10.01	16.5 ± 5.93
	*P*	0.004	0.02

MAP, mild acute pancreatitis; NLR, neutrophil‐to‐lymphocyte ratio; SAP, severe acute pancreatitis; WBC, white blood cell.

### 3.3 Values of WBC and NLR in predicting SAP by ROC curve

**Figure 1:**
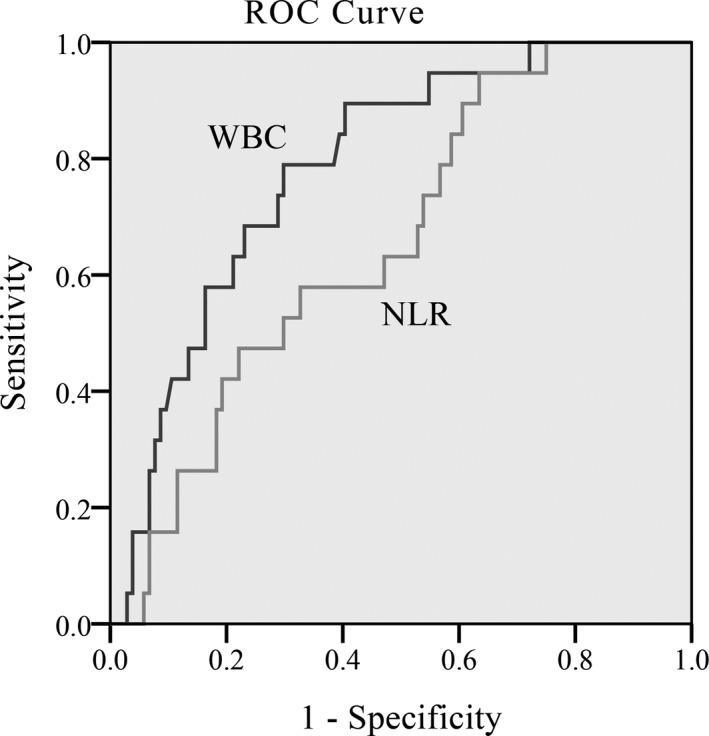
Receiver operating characteristic curves of WBC and NLR with respect to prediction of gallstone‐induced SAP. NLR, neutrophil‐to‐lymphocyte ratio; SAP, severe acute pancreatitis; WBC, white blood cell

**Figure 2:**
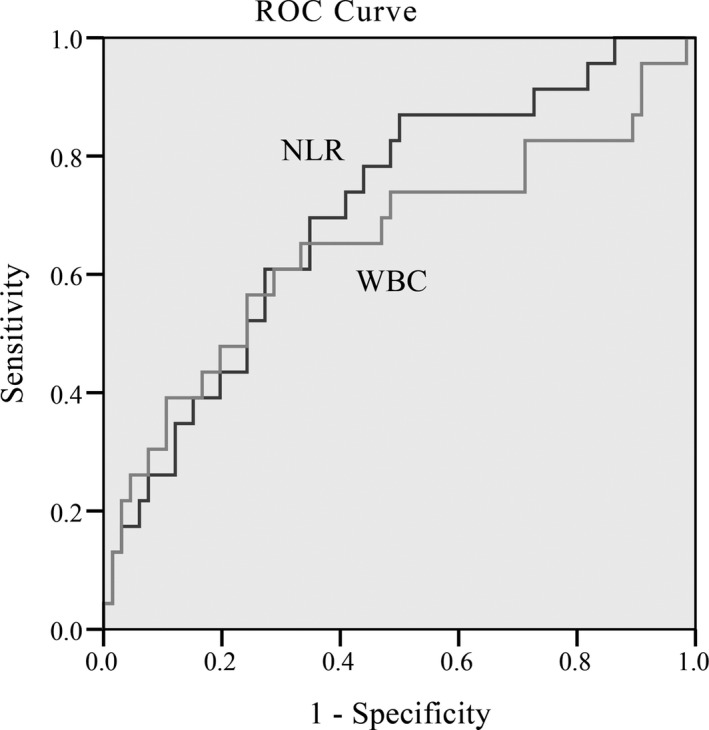
Receiver operating characteristic curves of WBC and NLR with respect to prediction of hyperlipidemia‐induced SAP. NLR, neutrophil‐to‐lymphocyte ratio; SAP, severe acute pancreatitis; WBC, white blood cell

We calculated the AUCs of NLR and WBC for predicting SAP by ROC curve in gallstone group and HTG group. For the gallstone AP, AUC value of NLR and WBC was 0.657 and 0.786, respectively. The best WBC cutoff value on predicting SAP in gallstone AP patients was 12.81 × 10^9^/L, with sensitivity and specificity of 78.9% and 70.2% (Figure 1). For the HTG‐AP group, AUC value of NLR and WBC was 0.706 and 0.653, respectively. The differences were both statistically significant. It seemed that NLR was better than WBC in predicting the severity of HTG‐AP. According to the ROC curve, the best cutoff value for NLR and WBC to differentiate HTG‐SAP with HTG‐MAP was more than 5.88 and 15.89 × 10^9^/L, respectively, with sensitivity and specificity of 87% and 50% for NLR and 56.5% and 75.76% for WBC (Figure 2). Thus, we came to conclusion that NLR was more sensitive and accurate in judging the severity of HTG‐AP, while WBC was more suitable for gallstone AP.

## 4 DISCUSSION

It was reported that there was a decrease in lymphocyte numbers and an increase in lymphocyte apoptosis, associated with lymphocyte dysfunction.^13^ NLR integrates two opposing and complementary components of the immune pathway and represents the balance between inflammatory activating factor neutrophils and inflammatory regulatory factor lymphocytes. Besides, the higher NLR value represents a more imbalanced inflammatory state.^14^ It was first reported as a parameter assessing systemic inflammation which could be easily measured by Zahorec.^15^ The course of SAP is complicated, and the prognosis is poor. Imbalance of immune response is one of the most important causes leading to severe pancreatitis.^16^ Therefore, we assumed that NLR should play an important role in predicting the severity of AP.

A few studies have investigated the relationship between NLR and outcome in patients with AP. Abaylı et al^17^ analyzed 435 patients with AP and found that NLR was greater in the group with a Ranson score ≥3 than the group with a Ranson score <3; thus, he concluded that NLR was a simple, practical, and effective marker for diagnosis of AP severity compared with current scoring systems. O'Connell et al^18^ revealed that NLR above five increased the risk of ICU admission. Another retrospective study revealed that the optimal cutoff value for NLR was 4.76 in predicting severity and 4.88 in predicting organ failure in acute pancreatitis.^19^ These results were close to our study, in which the optimal cutoff value for NLR in predicting HTG‐AP severity was 5.88.

Although there were some data regarding the predictive value of NLR in the prognosis of AP in the previous study, there was a lack of data regarding their utilities in specific causes of pancreatitis. Cho et al^20^ further reported that NLR and PLR could predict the severity of gallstone AP rather than alcoholic AP. However, the study was limited by enrollment of patients only with etiology of gallstone or alcohol. Wang et al^21^ retrospectively reviewed 110 patients with HTG‐AP and concluded that NLR represents an inexpensive, readily available test with a promising value to predict disease severity in HTG‐AP. This was consistent with our results.

In our study, we firstly investigated and compared the value of NLR and WBC in predicting AP severity in three common causes induced AP. When all AP cases were combined, NLR and WBC levels in patients with SAP were significantly higher than those in the MAP group. This was consistent with the previous study. However, after subgrouping AP by etiology, NLR was only predictive for SAP in the HTG‐AP group. On the contrary, WBC was an effective predictor for disease severity in both gallstone AP and HTG‐AP. Interestingly, we found that WBC and NLR did not differ between MAP and SAP in alcoholic AP. This suggested that WBC and NLR failed to assess the severity of inflammation in alcoholic AP, and other parameters were required for prediction. Azab et al^22^ reported that NLR was superior to WBC in predicting adverse outcomes of acute pancreatitis. However, our findings pointed out that NLR was more sensitive and accurate than WBC only in judging the severity of HTG‐AP. According to the ROC curve, the best cutoff value for NLR and WBC to differentiate HTG‐SAP with HTG‐MAP was more than 5.88 and 15.89 × 10^9^/L, respectively, while the best WBC cutoff value on predicting SAP in gallstone AP patients was 12.81 × 10^9^/L.

Compared with pancreatitis caused by other etiologies, HTG‐AP was more likely to have severe systemic inflammatory response syndrome and a poor prognosis.^23^ Zhu et al^12^ found that severe hyperlipidemic pancreatitis had significantly higher mortality rate than severe biliary pancreatitis. So previous studies suggested that HTG‐AP was more closely related to inflammation and inflammatory imbalance. Neutrophils promoted the spread of inflammation via secretion a series of inflammatory cytokines (interleukin‐1, interleukin‐6), proteolytic enzymes (myeloperoxidase, elastase, collagenase), and reactive oxygen species.^24^ It was reported that higher NLR value represented a more imbalanced inflammatory state.^14^ Therefore, our results which showed NLR could selectively predict the severity of HTG‐AP and was more sensitive and accurate than WBC in judging the severity can be explained as NLR could be regarded as a representative of imbalanced inflammatory cascade reaction. Because numbers of patients with different causes of AP were small, and data were from single center, the study on the role of NLR and WBC in the process of the severity of AP needs a larger sample and a more reasonable research plan.
